# Distinctive lung cancer incidence trends among men and women attributable to the period effect in Shanghai: An analysis spanning 42 years

**DOI:** 10.1002/cam4.2917

**Published:** 2020-02-19

**Authors:** Li Xie, Ying Qian, Yishan Liu, Yixuan Li, Sinong Jia, Herbert Yu, Chunfang Wang, Biyun Qian, Pingping Bao

**Affiliations:** ^1^ Hongqiao International Institute of Medicine Shanghai Tong Ren Hospital and Clinical Research Institute Shanghai Jiao Tong University School of Medicine Shanghai People’s Republic of China; ^2^ School of Public Health Shanghai Jiao Tong University School of Medicine Shanghai People’s Republic of China; ^3^ Shanghai Municipal Center for Disease Control and Prevention Shanghai People’s Republic of China; ^4^ Cancer Epidemiology Program University of Hawaii Cancer Center Honolulu HI USA

**Keywords:** age‐period‐cohort model, gender disparity, incidence, Lung cancer, mortality

## Abstract

**Background:**

Many previous studies reported secular trend of lung cancer incidence and mortality, but little is known about the possible reasons for these trends.

**Methods:**

Data were obtained from Shanghai Cancer Registry. Age‐standardized rates were calculated and average annual percent changes (AAPCs) were evaluated by Joinpoint regression. Age, period, and birth cohort effects were assessed by age‐period‐cohort models.

**Results:**

From 1973 to 2010, compared with long‐time slowly increasing trend in women, male lung cancer incidence had significantly decreased between 2001 and 2009. After that lung cancer incidence rising sharply in women (AAPC = 14.13%, 95%CI: 2.68%‐26.86%, *P* = .016) and similar rising trends without statistical significance in men (AAPC = 2.96, 95%CI: −2.47%‐8.69%, *P* = .281) between 2010 and 2014. Age‐period cohort model showed the different patterns of period effects for lung cancer incidence between men and women. The period effects for lung cancer incidence showed rising effect for women, whereas there was decline effect for lung cancer incidence for men. On the other hand, the model showed a significant period effect in both genders with a similar fashion in mortality, yielding steady falling trends during the entire study period.

**Conclusions:**

The distinctive patterns of lung cancer incidence between men and women may be attributable to significant period effects, which reflected the changes in public health policies or diagnostic practices and highlighted the urgent of continued monitoring of gender‐specific risk factors for lung cancer incidence.

## Graphical abstract

This study found that distinctive patterns of lung cancer incidence between male and female may be attributable to significant period effects, which reflect the effectiveness of primary prevention measures and highlighted the urgent of continued monitoring of gender‐specific risk factors for lung cancer incidence.

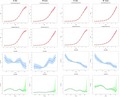

## 1 BACKGROUND

China is inhabited by one‐fifth of the world's population and contributes to almost 40% of all the lung cancer new diagnoses and deaths worldwide in 2018.^1^ Lung cancer becomes the most common among cancers in men and ranks only second to breast cancer in women; while lung cancer is the leading cause of deaths for both genders in 2015.^2^ The incidence and mortality rates of lung cancer in China are higher than the worldwide average, imposing an enormous disease burden and presenting a significant public health issue in China.^3^

Many previous studies reported distinctive patterns incidence of lung cancer between men and women, such as recent sharply increasing trends of female lung cancer incidence worldwide.^4^, ^5^, ^6^, ^7^ Dissimilar to those observed trends among women in Western European countries,^1^ the rising lung cancer incidence rates in Chinese women, despite their very low smoking prevalence, are considered to reflect exposures to environmental risk factors,^8^, ^9^ as well as the increasing use of low‐dose spiral computerized tomography (LDCT) screening.^7^ However, there is a lack of studies examined the possible reasons for different patterns of lung cancer incidence between men and women.

By using the long‐term cancer registry data in urban Shanghai, we conducted population‐based study, spanning 42 years, to explore the reasons for different patterns of lung cancer incidence by investigating the birth cohort effect (commonly a proxy for increased exposures to environmental risk factors, such as smoking and air pollution) and period effects (a surrogate for changes in public health policies or diagnostic practices, such as tobacco control policies).

## 2 MATERIALS AND METHODS

### 2.1 Data sources

Lung cancer incidence and mortality data were obtained from the Shanghai Cancer Registry. Details of the cancer registry data have been previously described.^10^, ^11^ Briefly, complete incidence and mortality data are available from 1973 onward. Shanghai Cancer Registry in the population of the urban areas covering 289.4 km^2^ and an average of 7 million residents have consistently reached the standards set by the International Agency for Research on Cancer (IARC), and have been published in its quinquennial publications.^12^ Lung cancer cases were identified by *The International Classification of Diseases* (ICD‐10) code range from C33 ~ C34. Each lung cancer case was reported by using a standardized malignant tumor case report card by all the 190 hospitals that are capable of diagnosis of malignant tumors in Shanghai. In 2014, 72.33% of lung cancer incidence were pathologically proven, 13.12% were radiological diagnosis, 4.51% through biochemical immunity, and nearly 10% were diagnosed through clinical diagnosis.^7^ The percentage of cancer cases identified by death certification only (DCO%) is very low, which ranged from 1.5% to 0.5% between 2005 and 2015. Annual population data during the study period were provided by the Department of Vital Statistics, Shanghai Municipal Center for Disease Control and Prevention.

### 2.2 Statistical methods

In this study, we provided a sketchy description for lung cancer incidence and mortality changes at first, including the number of newly diagnosed cases and deaths of lung cancer per year, crude annual incidence and mortality rates, and age standardized rates. Incidence and mortality data were split into the number of cases by gender subgroups and 5‐year age subgroups (0‐4, 5‐9, 10‐14, 15‐19, 20‐24, 25‐29, 30‐34, 35‐39, 40‐44, 45‐49, 50‐54, 55‐59, 60‐64, 65‐69, 70‐74, 75‐79, 80‐84, and ≥85) based on their age of diagnosis. Annual incidence and mortality for each gender and for each 5‐year age group were calculated: cases to 100 000 people per year. Annual age‐standardized rates (ASRs) per 100 000 men and women or both genders were calculated using the direct standardization method based on Segi's World Standard Population (1960).^13^ To further characterize trends in lung cancer incidence and mortality rate over time, the average annual percent changes (AAPC) and the corresponding 95% confidence intervals (CI) were calculated using Joinpoint Regression Program (version 4.5.0.1, National Cancer Institute).

The observed trends in incidence and mortality rates vs birth cohort and period by age were graphically presented using a semi‐log plot. The basic form of the APC model is as follows:

$$M_{\mathrm{ij}}=D_{\mathrm{ij}}|P_{\mathrm{ij}}=\mu +\alpha_{i}+\beta_{j}+\gamma_{k}+\delta_{\mathrm{ij}}$$

*M* corresponds to the incidence or mortality rate as a function of age and calendar period, meanwhile *D* corresponds to the lung cancer cases and deaths, and *P* corresponds to the population. *α_i_*, *β_j_*, and *γ_k_* means the associated age effect estimates, period effect estimates, and cohort effect estimates, respectively. *δ_ij_* is the random error, normally distributed.^14^ Data were aggregated cases and population for 13 five‐year age groups (20‐24 years to 80‐84 years) and 8 five‐year periods (ie, 1975‐1979 through 2010‐2014). Cohort effects by gender are presented as rate ratios (RRs), adjusted for age, with the 1940 birth cohort as reference. Hence, rate ratios less than 1.0 for a specific birth cohort indicate lower lung cancer incidence/mortality when compared with the 1940 cohort, whereas ratios greater than 1.0 indicate higher incidence/mortality. The goodness of fit for each model was estimated using the likelihood ratio test and the Akaike information criterion (AIC). The model analysis and presentation were performed using *apc* package in R (version 3.5.1).

## 3 RESULTS

### 3.1 Overall trends of lung cancer incidence and mortality

**Figure 1:**
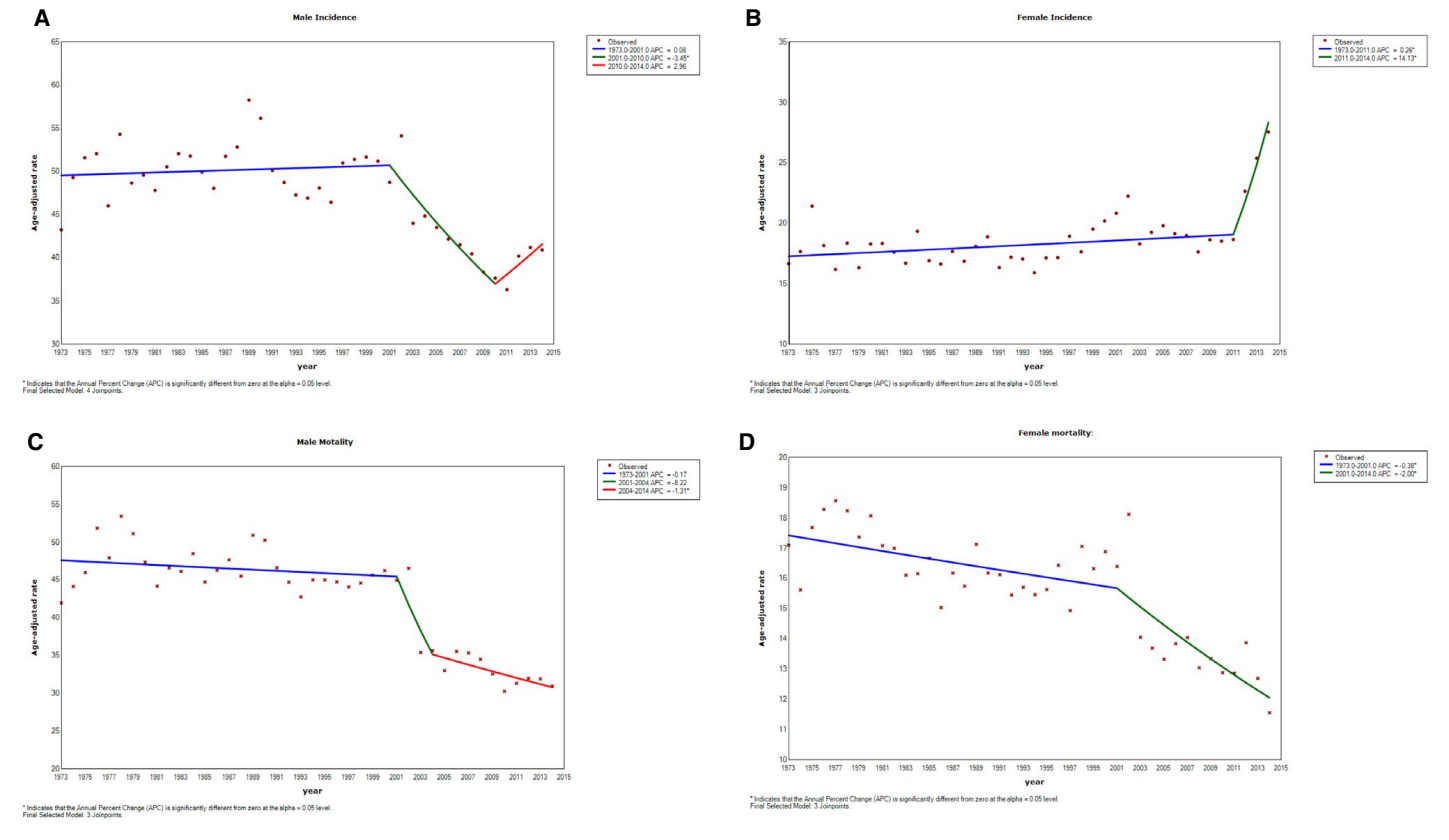
Joinpoint analysis of trends in age‐standardized (Segi's world standard population) incidence and mortality rates from lung cancer in both gender in Shanghai from 1973 to 2014. A, Male incidence; B, Female incidence; C, Male mortality; D, Female Mortality

From 1973 to 2001, lung cancer incidence in men was relatively stable with no significant change (AAPC = 0.08%, 95%CI: −0.20%, 0.37%, *P* = .558). Between 2001 and 2010, male lung cancer incidence decreased significantly (AAPC = −3.45%, 95%CI: −5.24%, −1.63%, *P* < .001). However, it is noteworthy that slightly increasing trends in male lung cancer incidence from 2010 to 2014 (AAPC = 2.96, 95%CI: −2.47%, 8.69%, *P* = .281). Different pattern was observed in female lung cancer incidence. From 1973 to 2011, female lung cancer was increased very slightly with an AAPC of 0.26% (95%CI: 0.04%, 0.48%, *P* = .022). However, there were a statistically significant sharp increased trends of female lung cancer after 2011 (AAPC = 14.13%, 95%CI: 2.68%, 26.86%, *P* = .016) (Figure 1).

Before 2001 years, lung cancer mortality was relatively stable with no significant change in men (AAPC = −0.17%, 95%CI: −0.42%, 0.09%, *P* = .201) and very slightly deceasing trends in women (AAPC = −0.38%, 95%CI: −0.66%, −0.10%, *P* = .009). While from 2001 to 2014, mortality rate of lung cancer showed a significantly decreasing trend in both men (2001‐2004: AAPC = −8.22%, 95% CI: −21.46% ‐ 7.26%, *P* = .271; 2004‐2014: AAPC = −1.31%, 95%CI: −2.50% ‐ −0.11%, *P* = .034) and women (AAPC = −2.00%, 95% CI: −2.87% ‐ −1.12%, *P* < .001) (Figure 1).

### 3.2 Time‐varying trends of lung cancer incidence and mortality by age and gender

We further stratified the population into categories according to gender and age at diagnosis. Interestingly, among the younger population, lung cancer incidence rates are higher among young women than among young men in under 40 age group (ASR women vs men: 4.51 vs 3.16/100 000), with the pattern confined to the 2008‐2014 period, whereas the mortality rate among the younger population similarly retained low rate (ASR women vs men:1.34 vs 1.53/100 000) (Table 1).

**Table 1:** Lung cancer incidence and mortality age‐specific rates and age‐standardized rates* (1/100 000) Shanghai (1973‐2014), by period

Age	Period								Total	Overall Change (%)
	1973‐1977	1978‐1982	1983‐1987	1988‐1992	1993‐1997	1998‐2002	2003‐2007	2008‐2014		
Incidence										
Men										
N	6752	8230	10 321	12 470	11 930	12 971	13 001	20 597	96 272	—
Age‐specific rates										
<40	4.23	3.56	3.76	4.26	4.77	4.9	3.25	3.16	4.05	−25.3
40‐49	24.40	23.99	19.40	14.36	16.38	23.72	25.41	24.01	21.62	−1.61
50‐59	108.22	98.25	101.40	99.65	68.67	67.87	70.00	92.33	88.82	−14.69
60‐69	300.06	296.76	284.98	307.38	276.49	258.05	179.53	172.31	248.99	−42.57
70‐69	393.60	467.36	502.30	539.55	510.47	587.4	479.64	379.34	479.10	−3.62
≥80	257.03	352.96	420.00	492.35	534.19	730.73	723.95	516.31	559.00	100.88
Age‐standardized rate	48.45	50.19	50.72	53.23	47.95	51.44	43.21	39.30	47.64	−18.89
Women										
N	2885	3352	4029	4737	4817	5861	6578	11 771	44 030	–
Age‐specific rates										
<40	4.16	2.9	3.07	3.55	2.97	3.33	2.51	4.51	3.37	8.41
40‐49	11.50	11.92	11.69	10.05	10.83	12.90	13.82	20.07	13.12	74.57
50‐59	39.95	37.20	34.63	36.23	33.67	38.98	33.44	48.42	39.41	21.2
60‐69	100.66	99.32	93.61	92.39	86.85	94.37	86.01	94.49	93.10	−6.13
70‐69	152.30	153.82	156.78	164.87	169.52	194.95	189.94	175.72	173.84	15.38
≥80	104.53	114.95	137.36	129.51	152.75	252.94	273.53	230.82	206.92	120.82
Age‐standardized rate	18.02	17.79	17.45	17.47	17.24	20.09	19.10	21.30	18.69	18.20
Mortality										
Men										
N	6289	7796	9442	11 186	11 179	11 699	10 870	17 692	86 153	—
Age‐specific rates										
<40	3.43	2.68	2.54	3.07	3.85	2.82	2.35	1.53	2.82	−55.39
40‐49	18.31	18.62	14.56	10.03	11.87	17.32	15.62	14.76	15.07	−19.41
50‐59	91.11	83.09	77.56	74.32	54.70	49.59	46.02	60.78	65.53	−33.29
60‐69	280.93	283.12	267.99	263.47	241.92	215.27	130.74	127.08	213.44	−54.77
70‐69	421.40	485.40	501.50	536.01	521.21	556.77	430.81	354.31	464.73	−15.92
≥80	356.65	419.95	426.43	556.67	581.37	800.89	735.35	585.95	613.21	64.29
Age‐standardized rate	46.37	48.54	46.66	47.60	44.31	45.59	34.98	31.92	42.71	−31.16
Women										
N	2700	3308	3741	4481	4556	5127	5142	8418	37 543	—
Age‐specific rates										
<40	2.60	2.68	1.73	2.18	2.08	2.08	1.59	1.34	2.00	−48.46
40‐49	8.74	11.07	9.13	6.77	7.87	9.13	7.55	8.77	8.54	0.32
50‐59	35.74	34.74	29.62	29.33	26.19	27.17	19.42	20.92	26.43	−41.47
60‐69	99.86	94.59	87.71	81.82	77.17	75.44	54.93	46.49	73.72	−53.45
70‐69	156.74	161.01	156.39	176.92	174.21	185.16	157.58	143.20	162.89	−8.64
≥80	123.54	141.21	139.05	157.05	175.86	255.38	270.61	251.33	221.31	103.45
Age‐standardized rate	17.45	17.55	16.03	16.13	15.64	16.96	13.79	12.90	15.67	−26.07

* Age standardized by Segi's world standard population 1960.

Over 42 years, male lung cancer incidence increased significantly only in 2 age groups (80‐84: AAPC = 1.3% and ≥ 85: APC = 2.2%); whereas in women, lung cancer incidence increased remarkably in all the age subgroups between 40 and 60 years old and over 80 years (AAPC range: 1.4%‐3.0%, *P* < .05) (Table 2). Mortality rate of lung cancer revealed a significantly declined trend in both men (AAPC = −0.8%, *P* < .01) and women (AAPC = −1.0%, *P* < .01) from 1973 to 2014.

**Table 2:** Age‐specific average annual percent change in incidence and mortality rate of lung cancer in Shanghai among men and women stratified by age at diagnosis from 1973 to 2014

Age (y)	Average annual percent change (%)											
	<40	40‐	45‐	50‐	55‐	60‐	65‐	70‐	75‐	80‐	≥85	Overall
Incidence												
Men	−0.7	0.2	−0.4	0.1	−0.3	−0.7	−1.1*	−0.8	0.2	1.3*	2.2*	−0.5
Women	2.2	2.3*	3.0*	1.8*	1.4*	0.7	0.2	0.2	0.7	1.9*	6.0*	1.0*
Total	−0.2	1.1	1.0	0.4	0.1	−0.3	−0.6	−0.4	0.8	1.8*	4.5*	0.1
Mortality												
Men	−4.1*	−3.0*	−1.0	−0.7	−1.3*	−1.7*	−2.1*	−1.0	0.4	1.0*	2.2*	−0.8*
Women	NA	−0.9*	−0.2	−1.2*	−1.9*	−2.1*	−2.1*	−1.0*	0.3	1.7*	5.2*	−1.0*
Total	−1.7*	−1.1	−1.2	−0.9*	−1.4*	−1.8*	−1.9*	−0.7	0.8	1.5*	3.7*	−0.7*

Abbreviation: NA, Not available.

* Indicate average annual percent change significantly different from 0 ( Two‐sided *P* < .05)

### 3.3 Age‐period‐cohort modeling analysis

**Figure 2:**
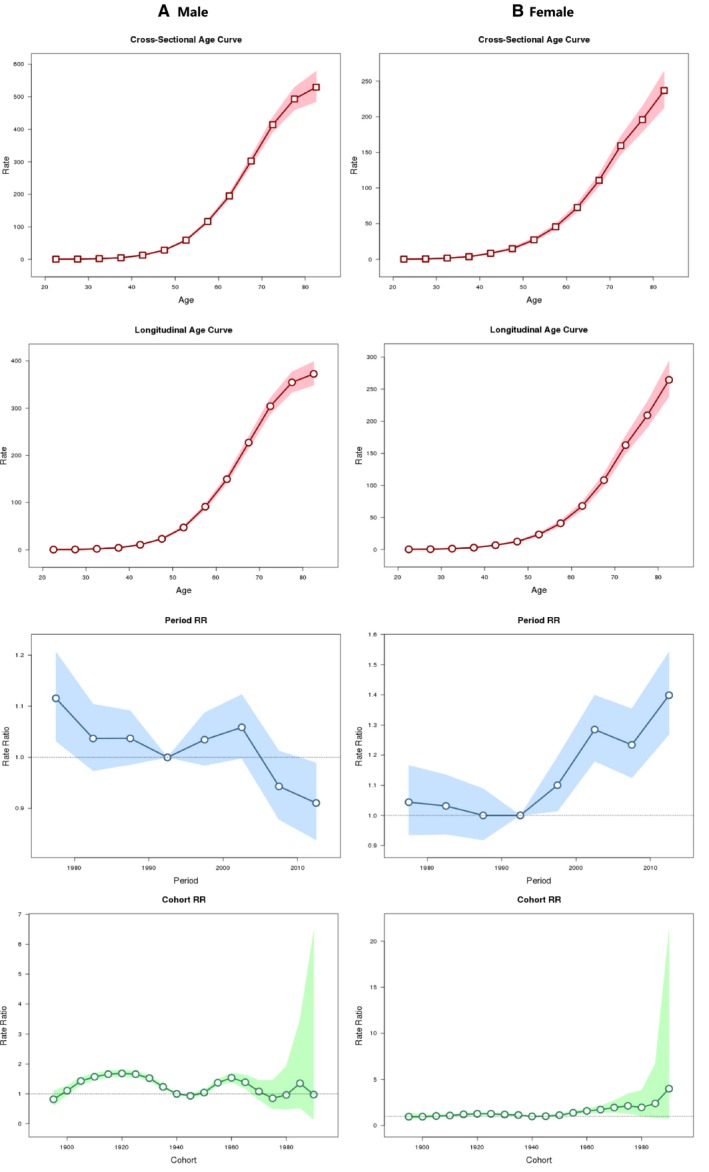
Age‐period‐cohort modeling for age‐standardized incidence rates for lung cancer in Shanghai (1973‐2014). A, Male incidence; B, Female incidence. Shaded bands indicate 95% confidence intervals. Dash lines indicate reference period (1990‐1994 as reference) and cohort (1940‐1944 birth cohort group as reference), respectively

Age‐period‐cohort modeling revealed significant period effects in lung cancer incidence, with the patterns for men and women distinctive from each other. For period effect on lung cancer incidence, using 1990‐1994 as reference, relative risks of lung cancer in men decreased until 1985‐1989, then increased until 2000‐2004, and decreased thereafter, whereas in women, relative risks slightly declined until 1990‐1994, then increased sharply with statistical significance after that, showing a different period effect pattern (Figure 2). Age effect of incidence rates of lung cancer was rising by increasing age in similar monotonic upward patterns for both genders but with a steeper slope for elderly women, peaking at 80‐ to 84‐year subgroup in both men and women. Using 1940‐1944 birth cohort group as reference, the model also showed flattening of risk of lung cancer incidence among recent cohorts in women, and a fluctuate risk in men.

**Figure 3:**
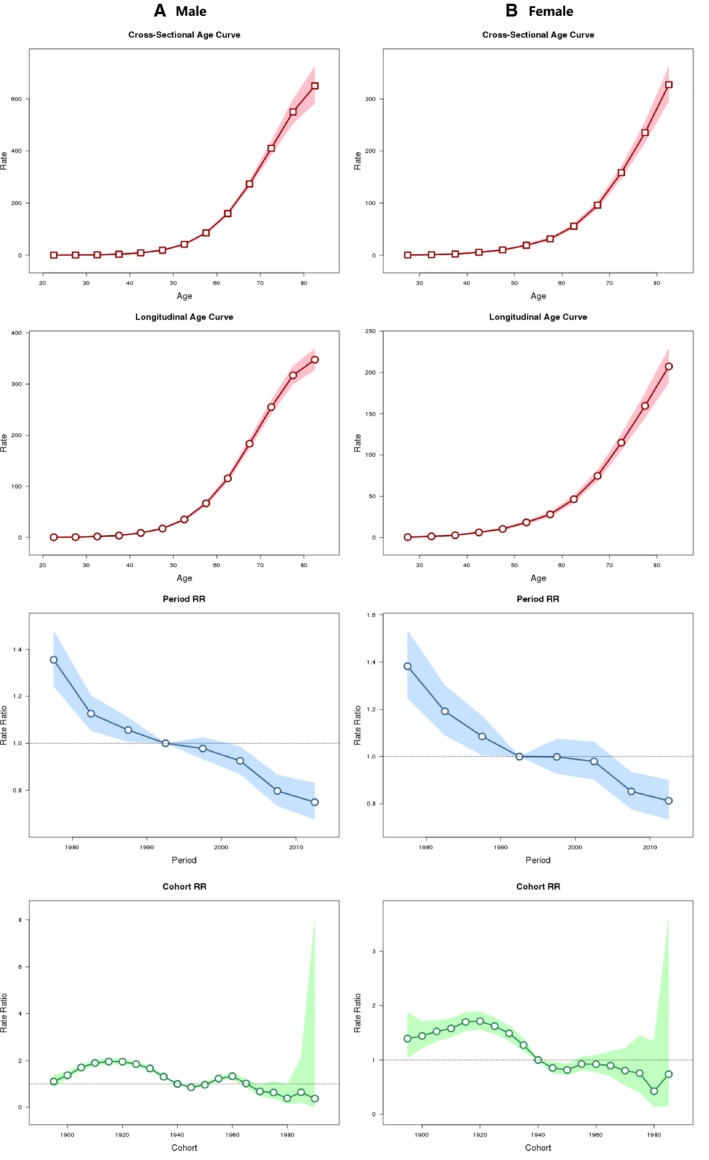
Age‐period‐cohort modeling for age‐standardized mortality rates for lung cancer in Shanghai (1973‐2014). A, Male mortality; B, Female mortality. Shaded bands indicate 95% confidence intervals. Dash lines indicate reference period (1990‐1994 as reference) and cohort (1940‐1944 birth cohort group as reference), respectively

For lung cancer mortality, adjusted by age and cohort, the model showed a significant period effect in both genders with a similar fashion, yielding significant steady falling transitions in mortality trends during the entire study period. The age and cohort effects were similar in both genders as well. Mortality rates of lung cancer were rising by increasing age and slightly declined by recent cohort in both genders (Figure 3).

## 4 DISCUSSION

Overall, by examining 42 years of IARC quality cancer registry data, our study confirms that different pattern of lung cancer incidence between men and women, and a similar decreasing trend of lung cancer mortality in both gender among the entire period in Shanghai. We did, however, observe a significant period effects in lung cancer incidence by age‐period‐cohort model analysis, with the patterns for men and women distinctive from each other.

The distinctive patterns for lung cancer incidence in men and women may attribute to significant period effects. The period effect was usually considered as a surrogate for changes in public health policies or diagnostic practices, such as tobacco control policies. A recent study conducted in United States found that comprehensive smoke‐free ordinances were associated with fewer new cases of lung cancer.^15^ As one of the earliest tobacco control cities in China, Shanghai Municipal Government released tobacco control policies *“Interim Provisions of Smoking Bans in Public Places in Shanghai”* in 1994.^16^ With the implementation of the tobacco control policies and the increasing public awareness of the hazards of smoking, the smoking rate decreased significantly among men (from 73.2% in 1997 to 48.5% in 2007).^17^, ^18^ The smoking rate was meager among women with less than 2% in Shanghai. In this study, we documented that the incidence rate of lung cancer among men decreased significantly from 2001 to 2010 with AAPC of −3.45% (*P* < .01), consistent with the findings that the smoking rate decreased significantly among men.

Our findings suggest that it is difficult to explain the recent rising trends in lung cancer incidence between men and women with the change in etiology, which may be related to the improvement of diagnostic methods and screening. The age‐period‐cohort model found that female lung cancer incidence rates significantly increased in recent years may attribute to the period effect as well. While the cancer screening factors driving the rising incidence in lung cancer have been discussed elsewhere,^7^ they found that lung cancer incidence in women rises significantly in urban Shanghai after introduction of low‐dose spiral computerized tomography (LDCT) screening^19^—the period effects on lung cancer incidence were ignored due to relatively short period (from 2005 to 2014) in their study. It is noteworthy that regardless of gender, we found a similar increasing trend of lung cancer incidence after 2011 in both men and women, which might have caused by the widespread use of LDCT as physical examinations among major hospitals in Shanghai.^20^, ^21^ In a recent study of lung cancer screening using low‐dose CT in asymptomatic population undergoing physical examination, Zhao et al reported that participation rate of women was higher than men (men vs women: 41% vs 59%).^22^ The difference in participation rates may contribute to the difference in the trends in incidence rates of lung cancer between men and women.

Another alternative explanation for the distinctive pattern of lung cancer incidence between men and women is that the association between environmental pollution and lung cancer risk. Dissimilar to those observed among females lung cancer incidence in several Western European countries, the prevalence of smoking among Chinese women did not increase substantially in recent years.^23^ Several epidemiological studies have reported that the significant association of air pollution and lung cancer mainly exist in nonsmokers.^24^, ^25^, ^26^ A recent spatiotemporal study found that the risks of lung cancer incidence related to a 10 µg/m increase in 2‐year average PM_2.5_ were higher in women (RR = 1.15, 95%CI = 1.12‐1.18) than men (RR = 1.06, 95%CI = 1.04‐1.07).^8^ Probably due to the majority of Chinese women had to take on the responsibilities of cooking, therefore more vulnerable exposed to cooking oil fume, which is likely to be a risk factor for lung cancer.^27^ Especially another study also found that women are more susceptible to lung cancer caused by air pollution.^28^ However, due to limited historical data collection of air pollution in Shanghai, there still needs further research to explore the association between air pollution and the incidence of lung cancer.

Among the younger population, lung cancer incidence rates are higher among young women than among young men, in which smoking prevalence is substantially lower. Lung cancer usually occurs in older patients, but there are many previous studies about lung cancer manifesting in younger patients.^29^ According to these studies, younger patients are more commonly nonsmoking women with an advanced stage. Many previous observational studies found that reproductive hormonal factors were associated with lung cancer risk.^30^, ^31^, ^32^ Also, these studies found exposure to menstrual and reproductive factors may play a role in the genesis of lung cancer in women, yet the mechanisms remain unclear.^33^ The potential biological mechanism for women being more sensitive to air pollution than men might be that the interaction effect of environmental factors and reproductive hormonal factors. Further studies collecting demographic, behavioral, and clinic data may help elucidate why women are likely to be more vulnerable than men.

Our study has some limitations. First, as an ecologic study, our analysis was unable to quantify the relative contributions of risk factors that may have resulted in the changes in incidence or mortality over study period. As known, cigarette smoking is one of the most crucial causal risk factors for the development of lung cancer. However, detailed information on cigarette smoking, alcohol drinking, and other known risk factors for lung cancer is not routinely captured by cancer registries records, so we were unable to directly measure the contribution of these risk factors to the distinctive pattern of incidence between men and women. However, the prevalence of smoking among women was lower than 2% and decreased slightly after the release of tobacco control policies. Second, the pathologic stages of lung cancer were not available due to the long period; this is a major limitation of this study. Thus, subgroup analyses and comparisons may not be feasible due to the unavailability or incompleteness of certain variables, such as stratified by squamous cell carcinoma and adenocarcinoma. Third, age‐period‐cohort modeling was used to examine age, period, and birth cohort effects. However, as calendar year minus age equals birth year, there is a perfect co‐linearity among age, period, and cohort effects, which makes it impossible to truly disentangle these effects.^34^ Fourth, the changes in the coverage of cancer registration during 30 years probably affect the results in both cancer incidence and mortality rates. Shanghai Cancer Registry was established in 1963, and complete incidence and mortality data have been collected since 1973 for urban areas and since 2002 for rural areas. Since Shanghai Cancer Registry has consistently reached the standards set by IARC and has been published in its quinquennial publications,^35^ results from SCR after 1973 would have high validity and good coverage of the population of Shanghai.

## 5 CONCLUSIONS

In summary, 42 years of cancer registry data indicated that distinctive patterns for lung cancer incidence between men and women may be attributable to significant period effects. While period‐specific effects may partly reflect the effectiveness of primary prevention measures, it also emphasize the urgency of continued monitoring of gender‐specific risk factors of lung cancer incidence.

## CONFLICT OF INTEREST

The authors have declared no conflicts of interest.

## AUTHOR CONTRIBUTIONS

Conceptualization: Li Xie, Ying Qian, Yishan Liu, Biyun Qian, and Pingping Bao; Data curation: Li Xie, Ying Qian, Yishan Liu, Yixuan Li, Sinong Jia, Herbert Yu, Chunfang Wang, Biyun Qian, and Pingping Bao; Formal analysis: Li Xie, Ying Qian, Yishan Liu, Biyun Qian, and Pingping Bao; Funding acquisition: Biyun Qian; Methodology: Li Xie, Ying Qian, Yishan Liu, Yixuan Li, Sinong Jia, Herbert Yu, Chunfang Wang, and Biyun Qian; Project administration: Biyun Qian; Resources: Biyun Qian; Software: Li Xie, Ying Qian, Yishan Liu, Biyun Qian, and Pingping Bao; Supervision: Biyun Qian and Pingping Bao; Validation: Li Xie, Ying Qian, Yishan Liu, Yixuan Li, Sinong Jia, Herbert Yu, Chunfang Wang, Biyun Qian, and Pingping Bao; Writing—original draft: Li Xie, Ying Qian, Yishan Liu, Biyun Qian, and Pingping Bao; Writing – review & editing: Li Xie, Ying Qian, Yishan Liu, Yixuan Li, Sinong Jia, Herbert Yu, Chunfang Wang, Biyun Qian, and Pingping Bao.

## Data Availability

Due to ethical approval reasons, we are unable to share data directly. However, most of data supporting the conclusions of this study are available in the Cancer Incidence in Five Continents (CI5) series: Cancer Incidence in Five Continents Volumes I to X by IARC (http://ci5.iarc.fr/CI5I-X/Default.aspx).
