# Supplementary figures and images for: Distinctive lung cancer incidence trends among men and women attributable to the period effect in Shanghai: An analysis spanning 42 years
Source: Cancer Med. 2020 Feb 19;9(8):2930–9. doi: 10.1002/cam4.2917 (PMC7163103; doi:10.1002/cam4.2917)

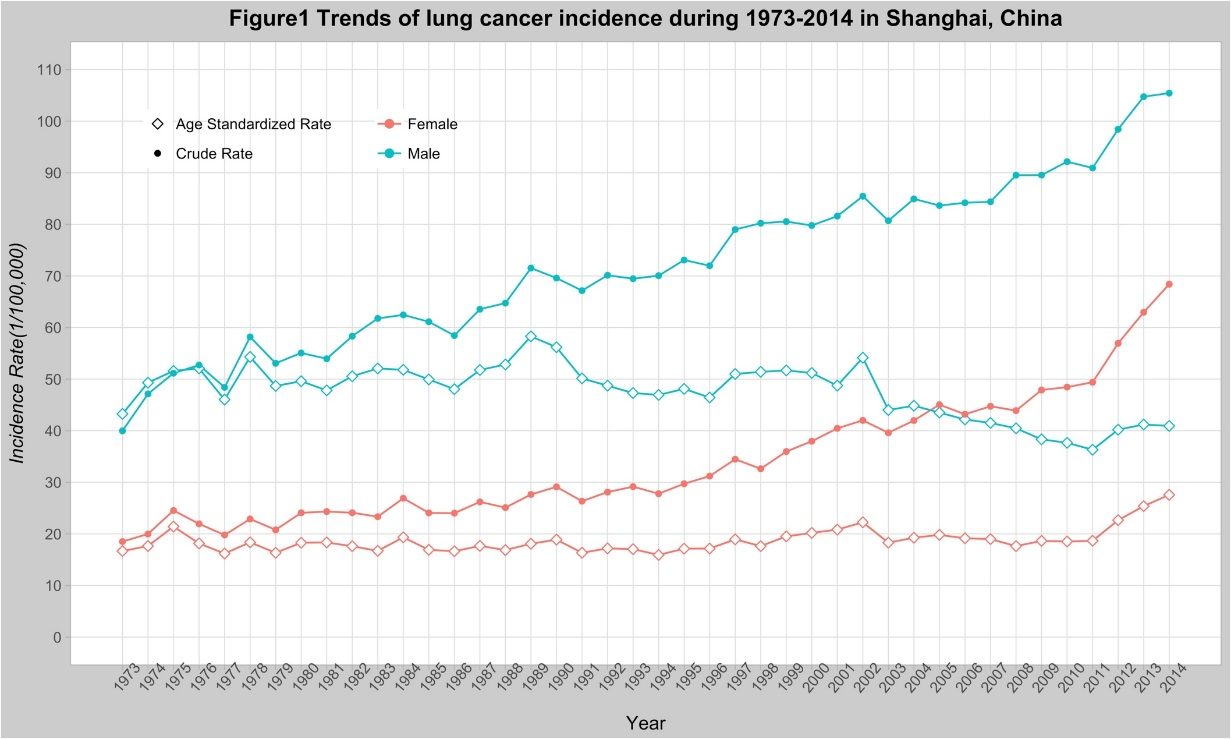

Supplement: Supplementary file 1 [file CAM4-9-2930-s001.tif]

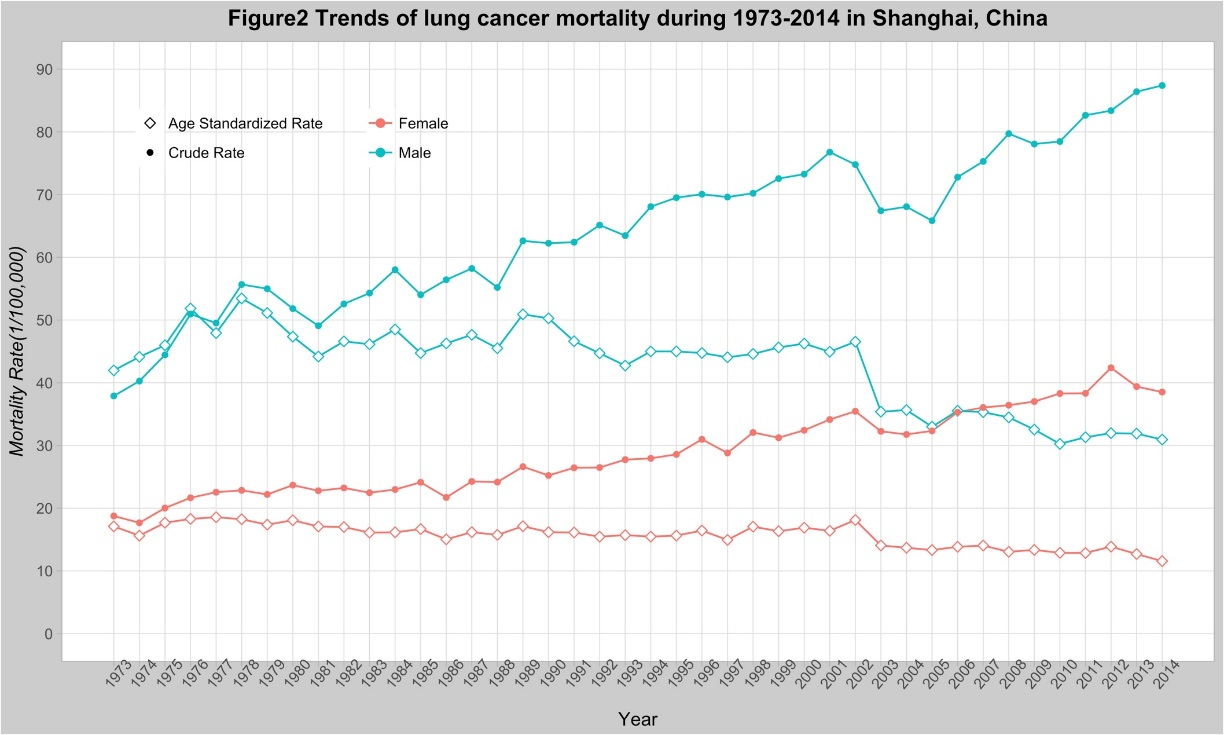

Supplement: Supplementary file 2 [file CAM4-9-2930-s002.tif]
