# Supplementary material for: Distinctive lung cancer incidence trends among men and women attributable to the period effect in Shanghai: An analysis spanning 42 years
Source: Cancer Med. 2020 Feb 19;9(8):2930–9. doi: 10.1002/cam4.2917 (PMC7163103; doi:10.1002/cam4.2917)
Supplement: Supplementary file 3 [file CAM4-9-2930-s003.docx]

**Table S1. Age-specific incidence rates by age group in both sexes, male and female, and their corresponding AAPCs by period**

| **Age／year** | **Total** |  | **Male** |  | **Female** |
| --- | --- | --- | --- | --- | --- |
| 40- | 1973-1992: -1.87* |  | 1973-1988: -2.76* |  | 1973-2009: 0.15 |
|  | 1992-1998: 7.31 |  | 1988-2005: 3.14* |  |  |
|  | 1998-2012: -1.50 |  | 2005-2012: -10.22* |  | 2009-2014: 19.19* |
|  | 2012-2014: 35.57 |  | 2012-2014: 43.48 |  |  |
| 45- | 1973-1995: -1.96* |  | 1973-1995: -3.11* |  | 1973-2010: 0.96* |
|  | 1995-2001: 9.10* |  | 1995-2001: 11.56* |  |  |
|  | 2001-2010: -1.26 |  | 2001-2014: -0.95 |  | 2010-2014: 24.13* |
|  | 2010-2014: 11.61* |  |  |  |  |
| 50- | 1973-1998:-1.72* |  | 1973-1988: -2.35* |  | 1973-2008: 0.04 |
|  | 1998-2014: 3.88* |  | 1988-2014: 4.07* |  | 2008-2014: 12.70* |
| 55- | 1973-2008:-1.49* |  | 1973-1991: -0.70 |  | 1973-2010: -0.46* |
|  |  |  | 1991-1996: -8.33* |  |  |
|  |  |  | 1996-2002: 3.11 |  |  |
|  | 2008-2014: 9.63* |  | 2002-2005: -9.84 |  | 2010-2014: 19.80* |
|  |  |  | 2005-2014: 6.71* |  |  |
| 60- | 1973-1990: -0.58 |  | 1973-1990: -0.20 |  | 1973-2010: -0.68* |
|  | 1990-2010:-2.43* |  | 1990-2010: -3.51* |  | 2010-2014: 14.81* |
|  | 2010-2014: 12.56* |  | 2010-2014: 11.82* |  |  |
| 65- | 1973-1999: -0.17 |  | 1973-1998: 0.12 |  | 1973-2010: -0.66* |
|  | 1999-2009: -4.98* |  | 1998-2009: -6.16* |  | 2010-2014: 8.49 |
|  | 2009-2014: 6.04* |  | 2009-2014: 4.30 |  |  |
| 70- | 1973-2002: 0.77* |  | 1973-1979: 3.94 |  | 1973-2014: 0.25 |
|  | 2002-2007: -7.24* |  | 1979-2000: 0.43 |  |  |
|  | 2007-2014:-0.03 |  | 2000-2014: -4.55* |  |  |
| 75- | 1973-1975: 16.37 |  | 1973-1983: 4.01* |  | 1973-2005: 1.64* |
|  | 1975-2004: 1.81* |  | 1983-2004: 1.26* |  | 2005-2014:2.74 |
|  | 2004-2014:-4.82* |  | 2004-2014: -5.71* |  |  |
| 80- | 1973-2002: 3.98* |  | 1973-2002: 3.83* |  | 1973-2005: 3.15* |
|  | 2002-2014:-3.15* |  | 2002-2014: -4.46* |  | 2005-2014:-2.23 |
| 85+ | 1973-1976: 34.04* |  | 1973-2004: 4.66* |  | 1973-1976: 62.88* |
|  | 1976-1996: 3.09* |  |  |  | 1976-1996: 0.83 |
|  | 1996-2002: 12.91* |  | 2004-2014: -4.93* |  | 1996-2001: 22.12 |
|  | 2002-2014:-3.31* |  |  |  | 2001-2014: -1.86 |

** Significantly different from 0 (P<0.05); AAPC = Average annual percent change; NA=Not available.*

**Table S2. Age-specific mortality rates by age group in both sexes, male and female, and their corresponding AAPCs by period**

| **Age／year** | **Total** |  | **Male** |  | **Female** |
| --- | --- | --- | --- | --- | --- |
| 40- | 1973-1978: 7.21 |  | 1973-1987: -5.21* |  | 1973-2014: -0.86* |
|  | 1978-1991:-4.87* |  | 1987-2001: 4.96 |  |  |
|  | 1991-1998: 8.22 |  | 2001-2014: -8.80* |  |  |
|  | 1998-2014:-4.29 |  |  |  |  |
| 45- | 1973-1978: 4.82 |  | 1973-1985: -0.86 |  | 1973-2014: -0.21 |
|  | 1978-1992:-4.53* |  | 1985-1993: -8.77* |  |  |
|  | 1992-2001: 5.76* |  | 1993-2001: 11.89* |  |  |
|  | 2001-2011:-0.62* |  | 2001-2014: 3.67* |  |  |
|  | 2011-2014:-16.48 |  |  |  |  |
| 50- | 1973-1996: -2.67* |  | 1973-1999: -2.86 |  | 1973-2014: -1.24* |
|  | 1996-2014: 1.33* |  | 1999-2014: 3.25* |  |  |
| 55- | 1973-2006: -2.34* |  | 1973-2005: -2.67* |  | 1973-2014: -1.85* |
|  | 2006-2014: 2.79 |  | 2005-2014: 3.86* |  |  |
| 60- | 1973-1998: -1.42* |  | 1973-1995: -1.07* |  | 1973-2014: -2.13* |
|  | 1998-2003: -10.78* |  | 1995-2005: -7.74* |  |  |
|  | 2003-2014: 1.76 |  | 2005-2014: 4.01* |  |  |
| 65- | 1973-1990: -0.70* |  | 1973-2000: -0.53* |  | 1973-1998: -1.23* |
|  | 1990-2005: -8.64* |  | 2000-2004: -14.02* |  | 1998-2014: -3.49* |
|  | 2005-2014: -0.83 |  | 2004-2014: -1.18 |  |  |
| 70- | 1973-1977: 6.13* |  | 1973-1978: 5.49* |  | 1973-1998: 0.15 |
|  | 1977-2002: -0.04 |  | 1978-2001: -0.00 |  |  |
|  | 2002-2005: -11.41 |  | 2001-2005: -9.73* |  | 1998-2014: -2.87* |
|  | 2005-2014: -1.89* |  | 2005-2014: -2.79* |  |  |
| 75- | 1973-1976: 14.02* |  | 1973-1976: 12.51* |  | 1973-2001: 1.56* |
|  | 1976-2001: 1.58* |  | 1976-2001: 1.46* |  | 2001-2014: -2.45* |
|  | 2001-2014: -3.59* |  | 2001-2014: -4.23* |  |  |
| 80- | 1973-2002: 3.26* |  | 1973-2002: 2.90* |  | 1973-2002: 2.67* |
|  | 2002-2014: -2.62* |  | 2002-2014: -3.55* |  | 2002-2014: -0.59 |
| 85+ | 1973-1975: 34.58 |  | 1973-2004: 3.93* |  | 1973-1976: 47.16 |
|  | 1975-1995: 2.19* |  |  |  | 1976-1983: -5.23 |
|  | 1995-2002: 11.18* |  | 2004-2014: -3.04 |  | 1983-2014: 4.25* |
|  | 2002-2014: -2.16 |  |  |  |  |

** Significantly different from 0 (P<0.05); AAPC = Average annual percent change; NA=Not available.*

**Figure S1. Trends of lung cancer incidence during 1973-2014 in Shanghai, China**

**Figure S2. Trends of lung cancer mortality during 1973-2014 in Shanghai, China**
